# Extracellular volume by dual-energy CT, hepatic reserve capacity scoring, CT volumetry, and transient elastography for estimating liver fibrosis

**DOI:** 10.1038/s41598-023-49362-0

**Published:** 2023-12-12

**Authors:** Mariko Mizuno, Kenichiro Tago, Masahiro Okada, Yujiro Nakazawa, Takayuki Arakane, Hiroki Yoshikawa, Hayato Abe, Naoki Matsumoto, Tokio Higaki, Yukiyasu Okamura, Tadatoshi Takayama

**Affiliations:** 1 Departments of Radiology, Nihon University School of Medicine, 30-1, Oyaguchikami-machi, Itabashi-ku, Tokyo, 173-8610 Japan; 2 Departments of Digestive Surgery, Nihon University School of Medicine, Tokyo, Japan; 3 Departments of Gastroenterology and Hepatology, Nihon University School of Medicine, Tokyo, Japan

**Keywords:** Hepatology, Liver diseases

## Abstract

Our purpose was to compare the efficacy of liver and splenic volumetry (LV and SV), extracellular volume (ECV) on dual-layer spectral-detector CT scoring systems for estimating liver fibrosis (LF) in 45 patients with pathologically staged LF. ECV measured on CT value (HU-ECV), iodine density (ID-ECV), atomic number (Zeff-ECV), and electron density (ED-ECV), LV or SV/body surface area (BSA), albumin bilirubin grade (ALBI), model for end-stage liver disease (MELD) score, aspartate aminotransferase platelet ratio index (APRI), and fibrosis index based on the four factors (FIB-4) were recorded. Transient elastography was measured in 22 patients, and compared to ECV. No correlation was found between transient elastography and all ECVs. Area under the curve (AUC) for estimating F4 on transient elastography was 0.885 (95% CI 0.745–1.000). ALBI was weakly associated with LF (*p* = 0.451), while MELD (*p* < 0.001), APRI (*p* = 0.010), and FIB-4 (*p* = 0.010) were significantly associated with LF. SV/BSA had a higher AUC than MELD, APRI, and FIB-4 for estimating F4 (AUC = 0.815, 95% CI 0.63–0.999), but MELD (AUC = 0.799, 95% CI 0.634–0.965), APRI (AUC = 0.722, 95% CI 0.561–0.883), and FIB-4 (AUC = 0.741, 95% CI 0.582–0.899) had higher AUCs than ALBI. SV/BSA significantly contributed to differentiation for estimating F4; odds ratio (OR) was 1.304–1.353 (Reader 1–2; R1–R2), whereas MELD significantly contributed to the differentiation between F0–2 and F3–4; OR was 1.528–1.509 (R1–R2). AUC for SV/BSA and MELD combined was 0.877 (95% CI 0.748–1.000). In conclusion, SV/BSA allows for a higher estimation of liver cirrhosis (F4). MELD is more suitable for assessing severe LF (≥ F3–4). The combination of SV/BSA and MELD had a higher AUC than SV/BSA alone for liver cirrhosis (F4).

## Introduction

In patients with liver tumors undergoing hepatectomy, severe liver fibrosis (LF) is problematic because it limits surgical treatment options and is associated with post-hepatectomy liver failure^1^. Moreover, liver cirrhosis (LC) increases the risk of hepatocellular carcinoma (HCC)^2^. The diagnosis of LF has relied on liver biopsy, but biopsy has limitations such as invasiveness, risk of complications, and sampling error^3^. Instead of invasive liver biopsy, non-invasive LF assessment is expected. It is based on modalities such as fibrosis markers, transient elastography, and magnetic resonance (MR) elastography^4^; there is no currently established LF assessment method. 

Imaging analysis of the liver parenchyma, which is where HCC originates, is important because prognosis depends not only on the tumor but also on the condition of the liver parenchyma (e.g., liver failure). There are several diagnostic challenges associated with the estimation of LF using CT, MR, or radioisotopes. MR elastography^5^ and computerized tomography volumetry (CTV) of the spleen^6^ have been reported as useful for estimating LF. Extracellular volume (ECV) is an index calculated using haematocrit (Hct), non-contrast phase CT, and contrast equilibrium phase CT. It is useful for estimating the degree of LF^7,8^. ECV requires measurement of CT values by placing regions of interest (ROIs)^6^, but ECV measured based on iodine density (ID) in dual-energy CT systems can stage LF^9,10^.

On the other hand, some scoring systems combine blood and other biochemical test data to assess liver function. Albumin bilirubin grade (ALBI), model of end-stage liver disease (MELD), aspartate aminotransferase to platelet count ratio index (APRI), and fibrosis index based on the four factors (FIB-4) index have been clinically used as non-invasive scoring systems in patients with liver dysfunction^11^.

Dual-layer dual-energy CT (DLCT) can simultaneously measure high- and low-energy projection data at the exact same spatial and angular location^12^. IQon spectral CT (Philips), which was used in our study, can be analysed retrospectively from spectral data, making it easy to perform post-examination studies that could not be envisaged at the outset. This postprocessing technique is used as necessary after CT examinations. DLCT enables quantification of the liver parenchyma using ID and ECV^9^, but the accuracy of effective atomic number (Effective Z; Zeff) and electron density (ED) analysis is unknown.

The purpose of this study was to compare the ability to predict LF of the following: CTV of the liver and spleen; ECV with ID; CT value, Zeff, and ED measured using DLCT; liver stiffness by transient elastography; and scoring systems that combine data from blood and other biochemical tests.

## Methods

### Patients

This study was approved by the institutional review board of Nihon University School of Medicine (RK-210413-9). The study complied with the Declaration of Helsinki. Informed consent was waived due to the retrospective nature of this study by our institutional review board (Nihon University Hospital, institutional review board, President Prof. Ishihara), but the opt-out was posted on the hospital's homepage. Consecutive patients who were candidates for liver resection to treat liver tumours had undergone preoperative quadri-phase CT. Patients who met the following criteria were excluded: (1) patients who could not be operated on due to liver dysfunction and poor patient performance status, although a CT scan was performed (Child–Pugh classification ICG-R15 ≥ 35% or serum total bilirubin level ≥ 2.0 mg/dL, tumour status, or portal hypertension including the presence of high-risk esophageal varices), and (2) quadri-phase CT < 3 months prior to liver resection. Between July 2021 and June 2022, 55 patients underwent liver dynamic CT before liver resection. Background liver disease, hepatic biochemical data, Child–Pugh score, and pathological fibrosis stage were obtained from the electronic medical records of our hospital.

### Scoring systems that combine blood and other biochemical test data

Liver function scores from the following scoring systems were calculated based on blood and other biochemical test data for all patients: ALBI^13^, MELD^14^, APRI^15^, and FIB-4 index^16^.

### CT examination

Four-phase dynamic liver CT was performed, which included the unenhanced phase, arterial phase (25 s from the 150 HU threshold of the aorta using bolus tracking), portal venous phase (50 s from start of bolus tracking), and equilibrium phase (160 s from start of bolus tracking). CT contrast agent (iomeprol; Iomeron® 350-syringe, Eisai, concentration: 350 mg I/mL, iodine content; 600 mg I/kg) was injected over 30 s intravenously at 1.9–5.0 mL/second via an antecubital vein. The DLCT scanner (IQon Elite Spectral CT, Philips) and scanning parameters and contrast agent injection protocols are shown in the Supporting material.

### Imaging analysis

#### DLCT parameters

Spectral-based imaging data were used for the analysis of ECV ID, Zeff, and ED in the liver at a workstation (IntelliSpace Portal version11; Philips Electronics). Two radiologists [M.M. (Reader 1, R1) and K.T. (Reader 2, R2)], each with 7 years of experience in body CT, independently placed ROIs in the left lateral, right anterior, and right posterior segments of the liver in the slice where the umbilical portion had the maximum cross-section.

#### Extracellular volume fraction (ECV) analysis

The mean ID of the liver parenchyma (ID-liver) for the left lateral, right anterior, and right posterior segments and the mean ID of the aorta (ID-aorta) were calculated based the equilibrium phase of the CT at a workstation. Image analysis was independently performed by two radiologists who were not aware of the liver biopsy results. A 1-cm ROI was drawn to prevent a peripheral liver zone of less than 1 cm. Values from three ROIs in the liver parenchyma were averaged. Because the equilibrium phase was taken approximately 3 min after contrast injection, it was necessary to exclude examinations in which the contrast timing for the liver parenchyma was too early. The CT value (E-aorta) of the aorta during the equilibrium phase and the CT value of the portal vein (E-portal) during the equilibrium phase of each patient were compared. Any significant differences (> ± 10 HU) between E-aorta and E-portal were considered outliers and excluded.

ECV for each parameter measured from DLCT, which includes CT value (HU-ECV), iodine density (ID-ECV), atomic number (Zeff-ECV), and electron density (ED-ECV), was calculated as follows:

$${\text{ECV}}\left( \% \right) = \left( {1 - {\text{haematocrit}}} \right) \times \left( {\Delta {\text{parameter}}_{{{\text{liver}}}} /\Delta {\text{parameter}}_{{{\text{aorta}}}} } \right) \times 100,$$

where Δparameter = difference between the unenhanced phase and the equilibrium phase (approximately 180 s).

#### CT volumetry (CTV) analysis

TLV and SV were automatically measured using a commercially available picture archiving and communication system (SYNAPSE VINCENT^®^, Fujifilm Medical). All CTV images were reconstructed from 1-mm–thick multiphasic liver CT sections. An abdominal radiologist with 7 years of experience (M.M.) semi-automatically generated CTV images using the SAI viewer with deep learning technology (SYNAPSE^®^, Fujifilm Medical). TLV and SV measurement was performed within approximately 1 min by SAI viewer with deep learning technology (SYNAPSE^®^, Fujifilm Medical). TLV included the volume of the hepatic tumours, but included the volumes of the intrahepatic and intrasplenic vessels to ensure uniformity and reproducibility of the liver. TLV and SV were corrected for body surface area (BSA), such as TLV/BSA, SV/BSA. BSA was calculated using Dubois’ formula (BSA [m^2^] = 0.007184 × height [cm]^0.725^ × weight [kg]^0.425^).

### Transient elastography measurements

Transient elastography with the M probe was performed as the abdominal ultrasonography examination within 6 months between examinations with CT. Twenty-two patients performed transient elastography. Liver stiffness was determined as the median value of ten measurements, except for the minimum and maximum values. Successful rates < 80% and interquartile ranges (IQR) > 40% were excluded. The correlation between transient elastography and HU-ECV, ID-ECV, Zeff-ECV, and ED-ECV was examined. ROC analysis was performed on the ability of transient elastography to estimate LF.

### Pathology

Pathological evaluation was performed for surgically resected specimens. Pathological fibrosis stage was evaluated by two pathologists using the New Inuyama Classification system^17^: F0, no fibrosis; F1, fibrous portal expansion; F2, bridging fibrosis; F3, bridging fibrosis with architectural distortion; and F4, cirrhosis.

### Statistical analysis

The Jonckheere–Terpstra test was used to determine whether there were significant trends in LF grading for CTV (spleen and liver), liver function scores (ALBI, MELD, APRI, and FIB-4 index) and ECV (Zeff, ED, CT value, and ID). Spearman rank correlation test was performed for the correlation between liver stiffness of transient elastography and each ECV. Receiver operating characteristic (ROC) analysis was performed according to each binary LF grading threshold. The area under the ROC curve (AUC) was calculated. AUCs for the candidate variables were compared using the Delong test. SPSS (Version 27) was used for analysis.

## Results

### Patients

Of 55 patients who underwent hepatic surgery, 10 patients were excluded because the interval between DLCT and surgery was longer than the duration specified in the inclusion criteria (n = 9) or image quality was poor (n = 1). Ultimately, 45 patients were included in the study. The characteristics of the 45 patients are shown in Table 1. In our study, hepatitis C virus (HCV) was the most common cause of liver disease (22.2%), followed by hepatitis B virus (HBV) (17.8%) and alcoholic cirrhosis (11.1%). Of 45 patients, 42 (93.3%) had a Child–Pugh score of 5 points and 2 patients (4.4%) had a Child–Pugh score of 6 points. One patient (2.2%) had a Child–Pugh score of 8 points.

**Table 1:** Patients characteristics.

Sex, n (%)		Laboratory data	
Male	35 (77.8%)	Hct (%), mean (SD)	42.4 (4.4)
Female	10 (22.2%)	AST (IU/L), mean (SD)	37 (19.6)
Age (yr), mean (SD)	67.2 (10.1)	ALT (IU/L), mean (SD)	34 (24.3)
BSA (m^2^), mean (SD)	1.68 (0.17)	Plt (10^9^/L), mean (SD)	20.4 (8.3)
Background liver disease, n (%)		INR, mean (SD)	1.05 (0.13)
HBV	8 (17.8%)	T-bil (mg/dL), mean (SD)	0.72 (0.30)
HCV	10 (22.2%)	Alb (g/dL), mean (SD)	3.9 (0.6)
Alcoholic liver disease	5 (11.1%)	Cr (mg/dL), mean (SD)	0.78 (0.29)
HCV + Alcoholic liver disease	2 (4.4%)	ICG-R15 (%), mean (SD)	10.86 (6.60)
HBV + HCV + Alcoholic liver disease	1 (2.2%)	ALBI, mean (SD)	-2.64 (0.51)
Others	19 (42.3%)	grade 1*, n (%)	26 (57.8%)
Child–Pugh score, n (%)		grade 2a*, n (%)	10 (22.2%)
5	42 (93.3%)	grade 2b*, n (%)	8 (17.8%)
6	2 (4.4%)	grade 3*, n (%)	1 (2.2%)
8	1 (2.2%)	MELD, mean (SD)	2.19 (5.18)
Pathological F grades, n (%)		APRI, mean (SD)	6.34 (3.92)
F0	11 (24.4%)	FIB-4, mean (SD)	24.04 (11.69)
F1	6 (13.3%)		
F2	12 (26.7%)		
F3	7 (15.6%)		
F4	9 (20.0%)		

BSA, body surface area; HBV, hepatitis B virus infection; HCV, hepatitis C virus infection; ALBI, albumin-bilirubin grade; MELD, model for end-stage liver disease score; APRI, aspartate aminotransferase-platelet ratio index, FIB-4, fibrosis index based on the four factors; Hct, hematocrit; AST, aspartate aminotransferase; ALT, alanine aminotransferase; Plt, platelet; INR, international normalized ratio; T-Bil, total bilirubin; Alb, albumin; Cr, creatinine; ICG-R15, indocyanine green retention rates at 15 min after injection.

*modified ALBI grade.

Twenty-two of 45 patients had transient elastography examinations within 6 months between ultrasonography and CT.

### CT volumetry, extracellular volume fraction (ECV), and scoring systems combining blood and other biochemical test data

#### Relationship between liver fibrosis and each parameter

**Figure 1:**
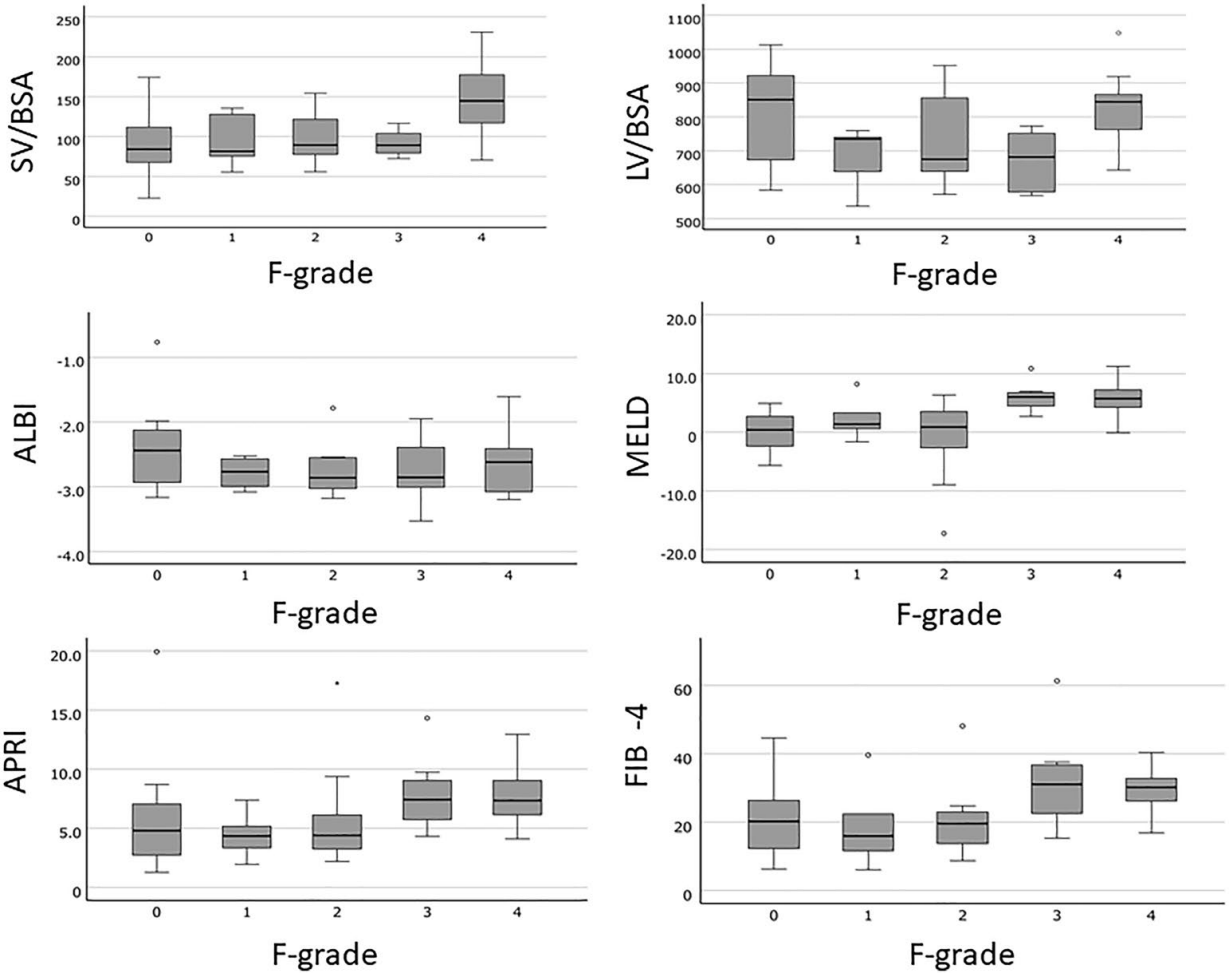
Correlation between CTVs or liver function tests and liver fibrosis. The box-and-whisker diagrams showed the correlation between imaging indices or liver function tests and liver fibrosis. SV/BSA, splenic volume/body-surface-area; LV/BSA, liver volume/body-surface-area; ALBI, Albumin-bilirubin-grade; MELD, model-for-end-stage-liver-disease-score; APRI, aspartate-aminotransferase-platelet-ratio-index; FIB-4, fibrosis-index-based-on-the-four-factors in the Fibrosis-4 score; F-grade, fibrosis grade.

**Figure 2:**
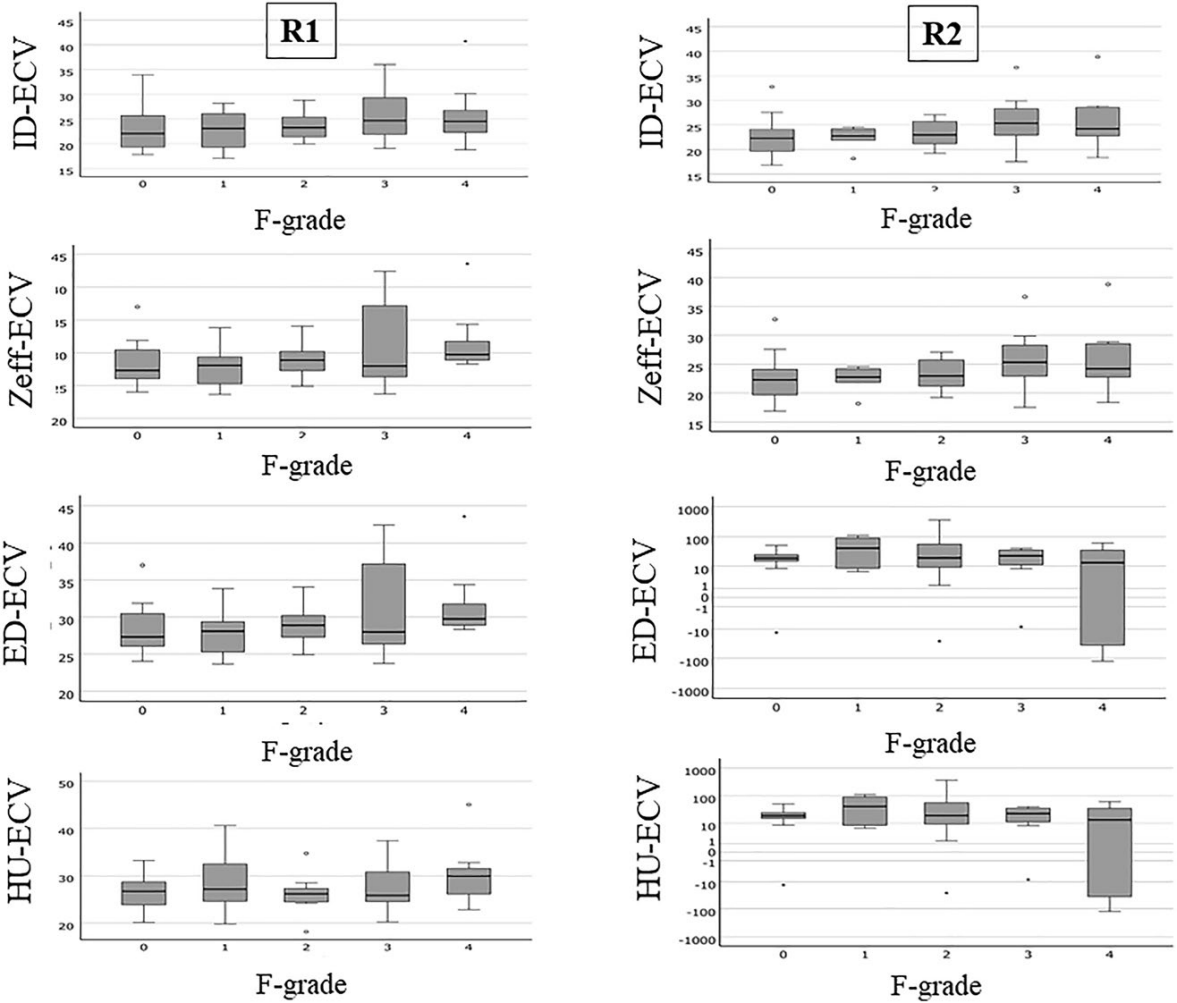
Correlation between ECV and liver fibrosis. The box-and-whisker diagrams showed the correlation between imaging indices and liver fibrosis. ID-ECV, ECV based on iodine density; Zeff-ECV, ECV based on atomic number; ED-ECV, ECV based on electron density; HU-ECV, ECV based on CT value; R1, Reader 1; R2, Reader 2.

Box-and-whisker diagrams show the correlation between imaging index or liver function test and LF (Figs. 1, 2). MELD and SV/BSA had relatively strong correlations with LF. For severe LF (≥ F3–4), SV/BSA, MELD, FIB-4 index, and Zeff-ECV were significantly correlated with severe LF (≥ F3–4) in the univariate analysis (Table 2) according to both R1 and R2 (Fig. 2). MELD was significantly correlated with severe LF (≥ F3–4) in the multivariate analysis (Table 2) according to both R1 and R2. SV, SV/BSA, LV, and MELD were significantly correlated with liver cirrhosis (F4) in the univariate analysis (Table 3) for LC (F4F4). SV/BSA and MELD were significantly correlated with liver cirrhosis (F4) in the multivariate analysis (Table 3) according to both R1 and R2.

**Table 2:** Univariable and multivariable analyses of predicting severe liver fibrosis (F3-4).

Variables	Univariable		Multivariable	
	OR (95%-CI)	*p* value	OR (95%-CI)	*p* value
R1				
SV^a^	1.136 (1.028–1.255)	0.013	–	–
SV/BSA^a^	1.199 (1.014–1.419)	0.034	1.048 (0.833–1.319)	0.690
LV^b^	1.177 (0.938–1.477)	0.160	1.070 (0.778–1.470)	0.678
MELD^c^	1.681 (1.219–2.319)	0.002	1.528 (1.066–2.191)	0.021
APRI^c^	1.179 (0.987–1.407)	0.069	–	–
FIB-4^c^	1.097 (1.023–1.176)	0.009	1.045 (0.952–1.147)	0.351
ID-ECV^c^	1.120 (0.980–1.280)	0.097	–	–
Zeff-ECV^c^	1.172 (1.003–1.370)	0.046	1.022 (0.843–1.239)	0.826
ED-ECV^c^	0.938 (0.862–1.022)	0.144	–	–
HU-ECV^c^	1.096 (0.968–1.240)	0.147	–	–
R2				
SV^a^	1.136 (1.028–1.255)	0.013	–	–
SV/BSA^a^	1.199 (1.014–1.419)	0.034	1.054 (0.836–1.328)	0.658
LV^b^	1.177 (0.938–1.477)	0.160	1.051 (0.965–1.146)	0.704
MELD^c^	1.681 (1.219–2.319)	0.002	1.509 (1.042–2.185)	0.030
APRI^c^	1.179 (0.987–1.407)	0.069	–	–
FIB-4^c^	1.097 (1.023–1.176)	0.009	1.044 (0.950–1.147)	0.371
ID-ECV^c^	1.70 (1.24–2.33)	0.048	–	–
Zeff-ECV^c^	1.239 (1.020–1.505)	0.031	1.036 (0.821–1.306)	0.767
ED-ECV^c^	0.856 (0.710–1.032)	0.103	–	–
HU-ECV^c^	1.217 (1.022–1.449)	0.027	–	–

R1, reviewer 1; R2, reviewer 2; OR, odds ratio; CI, confidence interval; SV, spleen volume; BSA, body surface area; LV, liver volume; MELD, Model for End-stage Liver Disease; APRI, aspartate aminotransferase-platelet ratio index, FIB-4, fibrosis index based on the four factors; ECV, extracellular volume; ID-ECV, ECV with Iodine density; Zeff-ECV, ECV with atomic number; ED-ECV, ECV with electron density ; HU-ECV, ECV with CT number

^a^Odds ratio for a 10 unit increase. Odds ratio for a 0.1 unit increase. ^b^Odds ratio for a 100 unit increase. Odds ratio for a 0.01 unit increase. ^c^Odds ratio for a 1 unit increase; 95%-CI, 95% confidence interval.

**Table 3:** Univariable and multivariable analyses of predicting liver cirrhosis (F4).

Variables	Univariable		Multivariable	
	OR (95%-CI)	*p* value	OR (95%-CI)	*p* value
R1				
SV^a^	1.221 (1.064–1.402)	0.005	–	–
SV/BSA^a^	1.412 (1.114–1.790)	0.004	1.304 (0.992–1.715)	0.057
LV^b^	1.414 (1.039–1.925)	0.028	1.181 (0.822–1.697)	0.368
LV/BSA^b^	1.582 (0.900–2.783)	0.111	–	–
MELD^c^	1.365 (1.057–1.763)	0.017	1.298 (0.969–1.738)	0.080
FIB-4^c^	1.051 (0.988–1.119)	0.113	–	–
Zeff-ECV^c^	1.119 (0.962–1.302)	0.146	–	–
HU-ECV^c^	1.126 (0.980–1.293)	0.093	–	–
R2				
SV^a^	1.221 (1.064–1.402)	0.005	–	–
SV/BSA^a^	1.412 (1.114–1.790)	0.004	1.353 (1.048–1.747)	0.020
LV^b^	1.414 (1.039–1.925)	0.028	–	–
LV/BSA^b^	1.582 (0.900–2.783)	0.111	–	–
MELD^c^	1.365 (1.057–1.763)	0.017	1.236 (0.920–1.662)	0.160
FIB-4^c^	1.051 (0.988–1.119)	0.113	–	–
ID-ECV^c^	1.114 (0.955–1.299)	0.17	–	–
Zeff-ECV^c^	1.164 (0.971–1.395)	0.101	–	–
ED-ECV^c^	0.811 (0.644–1.020)	0.074	–	–
HU-ECV^c^	1.177 (1.007–1.375)	0.041	1.088 (0.911–1.300)	0.349

R1, reviewer 1; R2, reviewer 2; OR, odds ratio; CI, confidence interval; SV, spleen volume; BSA, body surface area; LV, liver volume; MELD, Model for End-stage Liver Disease; APRI, aspartate aminotransferase-platelet ratio index, FIB-4, fibrosis index based on the four factors; ECV, extracellular volume; ID-ECV, ECV with Iodine density; Zeff-ECV, ECV with atomic number; ED-ECV, ECV with electron density ; HU-ECV, ECV with CT number.

^a^Odds ratio for a 10 unit increase. Odds ratio for a 0.1 unit increase. ^b^Odds ratio for a 100 unit increase. Odds ratio for a 0.01 unit increase. ^c^Odds ratio for a 1 unit increase, 95%-CI, 95% confidence interval.

MELD significantly contributed to the differentiation between F0–2 and F3–4. The odds ratio (OR) was 1.528 for R1 and 1.509 for R2. SV/BSA significantly contributed to the differentiation between F0–3 and F4. The OR was 1.304 for R1 and 1.353 for R2.

For the differentiation between F0 and F1–4, the AUC for MELD (0.717) was significantly higher than the AUC for ED-ECV (AUC, 0.533; *p* = 0.025) for R2 (Table 4). For the differentiation between F0–2 and F3–4, the AUC for MELD (0.877) was higher than the AUCs for SV/BSA (AUC, 0.672; *p* = 0.0264), LV (AUC, 0.619; *p* = 0.00806), LV/BSA (AUC, 0.504; *p* < 0.001), ALBI (AUC, 0.504; *p* = 0.00361), ID-ECV (AUC, 0.621; *p* = 0.0181) and (AUC, 0.675; *p* = 0.0436) for R1 and R2); Zeff-ECV (AUC, 0.651; *p* = 0.0228 and AUC, 0.683; *p* = 0.0317) for R1 and R2; HU-ECV (AUC, 0.619; *p* = 0.00908) for R1; and ED-ECV (AUC, 0.606; *p* = 0.0158) for R2. For the differentiation between F0–3 and F4, the AUC for SV (0.830) was higher than the AUCs for LV/BSA (AUC, 0.667; *p* = 0.0242) and ALBI (AUC, 0.534; *p* = 0.044) (Table 4).

**Table 4:** Liver fibrosis estimating ability of each imaging parameter

Variable	SV	SV/BSA	LV	LV/BSA
F0 vs. F1–4				
Optimal cutoff value	123.78	70.452	1446.14	865.935
Specificity[%]	0.636	0.364	0.545	0.455
Sensitivity[%]	0.912	0.941	0.824	0.882
AUC (95%-CI)	0.690 (0.466–0.914)	0.7626 (0.413–0.838)	0.529 (0.269–0.790)	0.642 (0.436–0.847)
*p*-value vs. MELD	0.802	0.407	0.290	0.596
F0–1 vs. F2–4				
Optimal cutoff value	123.78	89.147	1008.42	683.905
Specificity[%]	0.471	0.647	0.294	0.706
Sensitivity[%]	0.929	0.679	0.893	0.464
AUC (95%-CI)	0.674 (0.494–0.855)	0.647 (0.473–0.821)	0.521 (0.331–0.711)	0.536 (0.355–0.716)
*p*-value vs. MELD	0.813	0.615	0.121	0.161
F0–2 vs. F3–4				
Optimal cutoff value	167.65	96.764	1079.05	919.052
Specificity[%]	0.69	0.655	0.379	0.207
Sensitivity[%]	0.75	0.688	0.938	0.938
AUC (95%-CI)	0.735 (0.587–0.882)	0.672 (0.504–0.841)	0.619 (0.451–0.786)	0.504 (0.323–0.686)
*p*-value vs. MELD	0.070	0.026	0.008	< 0.001
F0–3 vs. F4				
Optimal cutoff value	175.41	103.49	1535.21	762.406
Specificity[%]	0.667	0.694	0.833	0.639
Sensitivity[%]	0.889	0.889	0.556	0.778
AUC (95%-CI)	0.830 (0.674–0.987)	0.815 (0.630–0.999)	0.731 (0.544–0.918)	0.667 (0.474–0.860)
*p*-value vs. SV	reference	0.581	0.150	0.024

Variable	ALBI	MELD	APRI	FIB-4
F0 vs F1–4				
Optimal cutoff value	− 2.936	5.325	3.158	12.704
Specificity[%]	0.636	1.000	0.364	0.364
Sensitivity[%]	0.824	0.382	0.912	0.912
AUC (95%-CI)	0.644 (0.425–0.864)	0.717 (0.558–0.875)	0.602 (0.381–0.822)	0.591 (0.383–0.799)
*p*-value vs. MELD	0.641	reference	0.283	0.189
F0–1 vs. F2–4				
Optimal cutoff value	− 2.544	4.238	5.315	12.704
Specificity[%]	0.471	0.882	0.647	0.353
Sensitivity[%]	0.750	0.536	0.643	0.964
AUC (95%-CI)	0.586 (0.410–0.762)	0.695 (0.537–0.854)	0.664 (0.494–0.834)	0.660 (0.485–0.834)
*p*-value vs. MELD	0.418	reference	0.707	0.682
F0–2 vs. F3–4				
Optimal cutoff value	− 3.077	2.698	6.382	26.117
Specificity[%]	0.862	0.724	0.793	0.828
Sensitivity[%]	0.312	0.938	0.688	0.688
AUC (95%-CI)	0.504 (0.313–0.696)	0.877 (0.771–0.984)	0.776 (0.640–0.912)	0.784 (0.647–0.922)
*p*-value vs. MELD	0.004	reference	0.138	0.155
F0–3 vs. F4				
Optimal cutoff value	− 2.645	3.627	5.315	26.117
Specificity[%]	0.583	0.694	0.556	0.750
Sensitivity[%]	0.667	0.889	0.889	0.778
AUC (95%-CI)	0.534 (0.291–0.777)	0.799 (0.634–0.965)	0.722 (0.561–0.883)	0.741 (0.582–0.899)
*p*-value vs. SV	0.044	0.777	0.374	0.423

Variable	ID-ECV (R1)	Zeff-ECV (R1)	ED-ECV (R1)	HU-ECV (R1)
F0 vs. F1–4				
Optimal cutoff value	22.313	27.907	37.8	22.829
Specificity[%]	0.545	0.545	0.500	0.273
Sensitivity[%]	0.706	0.735	0.765	0.912
AUC (95%-CI)	0.586 (0.362–0.809)	0.602 (0.394–0.809)	0.591 (0.373–0.810)	0.559 (0.358–0.760)
*p*-value vs. MELD	0.351	0.388	0.273	0.117
F0–1 vs. F2–4				
Optimal cutoff value	19.985	27.243	14.48	22.829
Specificity[%]	0.353	0.471	0.688	0.235
Sensitivity[%]	0.929	0.857	0.536	0.929
AUC (95%-CI)	0.601 (0.417–0.785)	0.645 (0.472–0.818)	0.683 (0.548–0.819)	0.536 (0.345–0.718)
*p*-value vs. MELD	0.406	0.646	0.340	0.127
F0–2 vs. F3–4				
Optimal cutoff value	24.551	27.907	9.283	28.667
Specificity[%]	0.655	0.448	0.786	0.793
Sensitivity[%]	0.562	0.875	0.625	0.562
AUC (95%-CI)	0.621 (0.443–0.798)	0.651 (0.473–0.828)	0.667 (0.484–0.851)	0.619 (0.437–0.800)
*p*-value vs. MELD	0.018	0.023	0.072	0.009
F0–3 vs. F4				
Optimal cutoff value	26.558	28.287	9.283	29.563
Specificity[%]	0.806	0.500	0.714	0.778
Sensitivity[%]	0.444	1.000	0.667	0.667
AUC (95%-CI)	0.608 (0.390–0.826)	0.698 (0.534–0.861)	0.606 (0.350–0.862)	0.676 (0.474–0.878)
*p*-value vs. SV	0.118	0.199	0.219	0.180

Variable	ID-ECV (R2)	Zeff-ECV (R2)	ED-ECV (R2)	HU-ECV (R2)
F0 vs F1–4				
Optimal cutoff value	22.355	22.274	35.2	27.437
Specificity[%]	0.545	0.455	0.889	0.909
Sensitivity[%]	0.735	0.853	0.412	0.412
AUC (95%-CI)	0.612 (0.400–0.824)	0.618 (0.425–0.810)	0.533 (0.351–0.714)	0.615 (0.425–0.805)
*p*-value vs. MELD	0.444	0.378	0.025	0.294
F0–1 vs. F2–4				
Optimal cutoff value	22.613	29.01	8.000	27.437
Specificity[%]	0.529	0.765	0.867	0.882
Sensitivity[%]	0.750	0.607	0.321	0.464
AUC (95%-CI)	0.643 (0.474–0.812)	0.658 (0.494–0.821)	0.564 (0.388–0.741)	0.651 (0.487–0.815)
*p*-value vs. MELD	0.639	0.711	0.199	0.656
F0–2 vs. F3–4				
Optimal cutoff value	22.613	31.76	− 8.333	27.845
Specificity[%]	0.483	0.931	0.926	0.862
Sensitivity[%]	0.875	0.500	0.312	0.625
AUC (95%-CI)	0.675 (0.500–0.849)	0.683 (0.500–0.866)	0.606 (0.424–0.789)	0.737 (0.571–0.903)
*p*-value vs. MELD	0.044	0.032	0.016	0.139
F0–3 vs. F4				
Optimal cutoff value	22.613	30.24	− 8.765	26.973
Specificity[%]	0.417	0.778	0.941	0.722
Sensitivity[%]	0.889	0.667	0.444	0.889
AUC (95%-CI)	0.636 (0.423–0.848)	0.673 (0.445–0.901)	0.647 (0.399–0.895)	0.784 (0.616–0.952)
*p*-value vs. SV	0.222	0.308	0.096	0.707

SV, splenic volume; BSA, body surface area; SV/BSA, ratio of SV to BSA; LV, liver volume; LV/BSA, ratio of SLV to BSA; ALBI, albumin-bilirubin grade; MELD, model for end-stage liver disease score; APRI, aspartate aminotransferase-platelet ratio index; FIB-4, fibrosis index based on the four factorsFibrosis-4 score; ID-ECV, ECV by iodine density; Zeff-ECV, ECV by atomic number; ED-ECV, ECV by electron density; HU-ECV, ECV by CT number; AUC, area under the ROC curve; R1, Reviwer 1; R2, Reviwer 2. AUCs are shown along with 95% confidence intervals (95%-CI).

**Figure 3:**
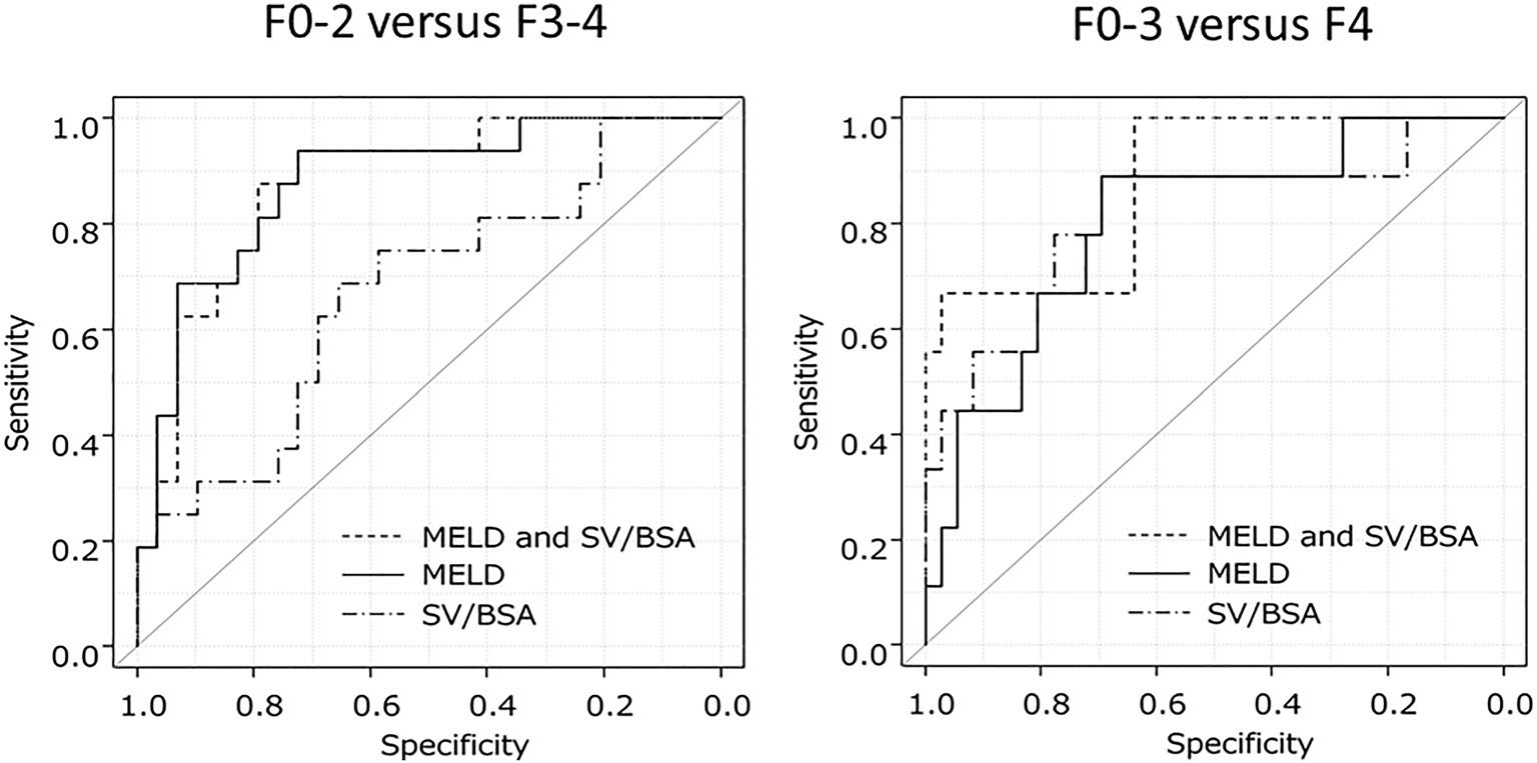
ROC analysis of MELD, SV/BSA, MELD + SV/BSA for identifying F3–4 or F4. For differentiating between F0–2 and F3–4, combining SV/BSA with MELD resulted in a poor increase in diagnostic accuracy according to the ROC analysis. Combining SV/BSA with MELD resulted in an increase in diagnostic performance for differentiating between F0–3 and F4. MELD, model-for-end-stage-liver-disease-score; SV/BSA, splenic volume/ body-surface-area.

For the differentiation between F0–2 and F3–4, combining SV/BSA with MELD resulted in a small increase in diagnostic accuracy according to ROC analysis, while combining SV/BSA with MELD to differentiate between F0–3 and F4 resulted in an increase in diagnostic performance (Fig. 3).

### Transient elastography measurement

The distribution of LF measured by transient elastography is F0, n = 2; F1, n = 2; F2, n = 9; F3, n = 5; and F4, n = 4. No correlation was found between transient elastography and HU-ECV (r = 0.451), ID-ECV (r = 0.249), Zeff-ECV (r = 0.249), or ED-ECV (r = -0.083). AUC for estimating F4 on US-elastography was 0.885 (95% CI = 0.745–1.000).

The AUCs to distinguish F1–4, F2–4, and F3–4 from F0, F0–1, and F0–2 were 0.588 (95% CI 0.312–0.864), 0.667 (95% CI 0.416–0.917), and 0.649 (95% CI 0.405–0.893) on transient elastography, respectively.

## Discussion

Our study sought to compare the ability to predict LF for the following: CTV of the liver and spleen; ECV with ID; CT value, Zeff, and ED measured using DLCT; liver stiffness by transient elastography, and scoring systems that combine data from blood and other biochemical tests. SV/BSA is a better predictor of LF F4(F4), whereas MELD is suitable for LF ≥ F3–4. Interestingly, the combination of SV/BSA and MELD had a higher AUC than SV/BSA alone for liver cirrhosis (F4). LF staging based on liver biopsy is difficult because of heterogeneous collagen deposition. However, resected liver specimens were analyzed for all study patients; pathological assessment is a reliable assessment of LF. IQon spectral CT, which was used in our study, can be performed retrospectively, making it easy to perform post-examination studies that could not be envisaged at the outset. Therefore, the assessment of ID, Zeff, and ED was performed with ECV, which was not found to be useful for the assessment of LF. ED reflects the probability of an electron being present at a specific location^18^. It has been reported to indicate cellularity^19^. The analysis of ED was shown to be useful for grading gliomas. However, ED-ECV and ID-ECV were not specific markers for LF in our present study. However, Zeff-ECV was significantly associated with LF in the univariate analysis for LF ≥ F3–4, but not in the multivariate analysis (Table 2). In the present study, none of the ECVs using parameters from DLCT were considered useful for estimating LF. However, Sofue et al.^10^ reported that ID-ECV is more strongly correlated with LF stage, and ID measurement with DLCT might be able to capture iodine retention in the hepatic parenchyma more accurately than conventional CT values (HU-ECV). HU-ECV for R2 was found to be significantly associated with LF in the univariate analysis for F4, but not in the multivariate analysis (Table 3). Tago et al. stated that SV/BSA and HU-ECV are better methods for estimating F4^6^. Therefore, our results are consistent with their analysis.

In our study, liver function scoring systems (ALBI, MELD, APRI, and FIB-4 index) were calculated from blood and other biochemical test data for all patients. These scoring systems are used to estimate the severity of LF without the need for a liver biopsy. While non-invasive methods such as ALBI, MELD, APRI, and FIB-4 index can provide an estimate of LF severity, they are not always accurate and might not reflect the true extent of fibrosis in all patients. We have shown that MELD can estimate LF. MELD is a scoring system that uses the values of three laboratory parameters (total bilirubin, creatinine, and INR) to estimate the severity of liver disease and predict short-term mortality in patients with LC. For liver disease, clinical assessment with laboratory tests, Child–Pugh score, and MELD should occur every 6 months^20^. In addition, we believe that splenic volume measurement (SV/BSA) with CT could be used to assess severe LF.

Transient elastography had a higher ability for estimating F4 (AUC = 0.885). However, transient elastography did not have a high ability to identify LF except for estimating F4 (AUC = 0.588–0.667). This trend was also seen for SV/BSA. Not only SV/BSA but also combining SV/BSA with MELD is useful to estimate cases of strong liver stiffness (Fig. 3). The estimation of LF by combining information from routine CT scans with hepatic reserve capacity scoring may be of equal value to that of transient elastography.

In addition to liver fibrosis, iron and fat deposits in the liver progress to chronic liver disease. Dual-energy CT based iron/fat decomposition algorithm accurately measured hepatic iron and fat when both were present in a rabbit model^21^. Even in the study of fat quantification of dual-energy CT by patients, the multimaterial decomposition algorithm quantifying hepatic fat in dual-energy CT images is accurate and reproducible^22^. It will be necessary to estimate the tissue of liver cirrhosis by using such techniques in combination with imaging.

Our study had several limitations. First, the number of patients who underwent both DLCT and liver surgery was small. There were also a small number of patients in each LF stage. At present, the use of dual-energy CT for liver disease is limited due to the number of units in use, but it will be necessary to increase the number of studies using similar dual-energy CT devices in the future. Second, selection bias was present because all patients were candidates for liver surgery. There was heterogeneity in liver disease etiology in our study. A multicenter prospective study that includes patients who undergo liver biopsy is recommended. Third, the equilibrium period used to calculate ECV (180 s after contrast injection) may not be an appropriate time, although it was used in terms of throughput in clinical CT examinations. In addition, ID-ECV has been examined in another study^10^ and its usefulness has been noted, but there is no study of ED-ECV, and it is unclear whether the use of ECV as a study of ED in LF was appropriate. Various additional studies will be needed in the future. Fourth, comparisons with transient elastography are limited, because of the small number of patients. Future studies with a large number of cases are needed to clarify.

In conclusion, SV/BSA allows for higher estimation of LC (F4), whereas MELD is more suitable for the assessment of severe LF (≥ F3–4). The combination of SV/BSA and MELD resulted in a higher AUC than SV/BSA alone for evaluating LC (F4). Although there is no universally validated clinical tool for predicting LF, combined assessment of MELD and SV/BSA is promising as a novel biomarker for estimating LF as accurately as possible. Overall, the limitations in assessing LF highlight the importance of using a combination of different methods, including MELD and SV, to accurately diagnose and manage LF.

### Supplementary Information

Supplementary Table 1.

## Data Availability

The data analyzed in this study is available from the corresponding author on reasonable request.

## Abbreviations

ALBI: Albumin bilirubin grade
ALT: Alanine aminotransferase
APRI: Aspartate aminotransferase platelet ratio index
AST: Aspartate aminotransferase
Alb: Albumin
AUC: Area under the ROC curve
BSA: Body surface area
CI: Confidence interval
CTV: Computerized tomography volumetry
DLCT: Dual layer spectral detector CT
ECV: Extracellular-volume
ED-ECV: ECV by electron density
FIB-4: Fibrosis index based on the four factors
HCC: Hepatocellular carcinoma
HBV: Hepatitis B virus infection
HCV: Hepatitis C virus infection
Hct: Hematocrit
HU: Hounsfield unit
HU-ECV: ECV by CT value
ICGR15: Indocyanine green retention rates at 15 min after injection
ID-ECV: ECV by iodine density
LC: Liver cirrhosis
LF: Liver fibrosis
LV: Liver volume
MELD: Model-for-end-stage-liver-disease-score
R1: Reader 1
R2: Reader 2
ROC: Receiver operating characteristic
ROI: Region of interest
SV: Splenic volume
Zeff-ECV: ECV by atomic number
95% CI: 95% Confidence interval
