# Supplementary material for: Extracellular volume by dual-energy CT, hepatic reserve capacity scoring, CT volumetry, and transient elastography for estimating liver fibrosis
Source: Sci Rep. 2023 Dec 12;13:22038. doi: 10.1038/s41598-023-49362-0 (PMC10716370; doi:10.1038/s41598-023-49362-0)
Supplement: Supplementary file 1 — Supplementary Table 1. [file 41598_2023_49362_MOESM1_ESM.docx]

**Supporting material.**

**Table 1**

CT Scanning parameters and contrast agent injection protocols

Platform IQon spectral CT

Tube voltage (KVp) 120

Tube current (mAs) Auto exposure control (AEC)

Helical Pitch 0.891

Detector collimation (mm) 64×0.625

Rotation time (s) 0.27

Iodine dose (mg/kg) 600

Bolus tracking trigger (HU) 150

Scan delay (s) 25(HAP) / 50(PVP) / 160(EP)

Injection duration (s) 30

Fractional dose (mgI/kg/s) 20

Image reconstruction spectral level 3

iDOSE level 2 (5mm slice)，level 3 (1mm slice)

Slice thickness (mm) 5mm,1mm (spectral image, 1mm)

Note;

HAP, hepatic arterial phase; PVP, portal venous phase; EP, equilibrium phase; iDOSE,

iterative model reconstruction low-dose
